# Co-Administration of a Plasmid DNA Encoding IL-15 Improves Long-Term Protection of a Genetic Vaccine against *Trypanosoma cruzi*

**DOI:** 10.1371/journal.pntd.0000983

**Published:** 2011-03-08

**Authors:** Christopher S. Eickhoff, Jose R. Vasconcelos, Nicole L. Sullivan, Azra Blazevic, Oscar Bruna-Romero, Mauricio M. Rodrigues, Daniel F. Hoft

**Affiliations:** 1 Department of Internal Medicine, Saint Louis University, Saint Louis, Missouri, United States of America; 2 Centro de Terapia Celular e Molecular, Escola Paulista de Medicina, Universidade Federal de São Paulo, São Paulo, Brazil; 3 Department of Molecular Microbiology, Saint Louis University, Saint Louis, Missouri, United States of America; 4 Departamento de Microbiologia, Instituto de Ciências Biológicas, Universidade Federal de Minas Gerais, Belo Horizonte, Brazil; New York University School of Medicine, United States of America

## Abstract

**Background:**

Immunization of mice with the *Trypanosoma cruzi trans*-sialidase (TS) gene using plasmid DNA, adenoviral vector, and CpG-adjuvanted protein delivery has proven highly immunogenic and provides protection against acute lethal challenge. However, long-term protection induced by TS DNA vaccines has not been reported. The goal of the present work was to test whether the co-administration of a plasmid encoding IL-15 (pIL-15) could improve the duration of protection achieved through genetic vaccination with plasmid encoding TS (pTS) alone.

**Methodology:**

We immunized BALB/c mice with pTS in the presence or absence of pIL-15 and studied immune responses [with TS-specific IFN-γ ELISPOT, serum IgG ELISAs, intracellular cytokine staining (IFN-γ, TNF-α, and IL-2), tetramer staining, and CFSE dilution assays] and protection against lethal systemic challenge at 1 to 6 months post vaccination. Mice receiving pTS alone developed robust TS-specific IFN-γ responses and survived a lethal challenge given within the first 3 months following immunization. The addition of pIL-15 to pTS vaccination did not significantly alter T cell responses or protection during this early post-vaccination period. However, mice vaccinated with both pTS and pIL-15 challenged 6 months post-vaccination were significantly more protected against lethal *T. cruzi* challenges than mice vaccinated with pTS alone (P<0.05). Improved protection correlated with significantly higher numbers of TS-specific IFN-γ producing total and CD8^+^ T cells detected>6 months post immunization. Also, these TS-specific T cells were better able to expand after in vitro re-stimulation.

**Conclusion:**

Addition of pIL-15 during genetic vaccination greatly improved long-term T cell survival, memory T cell expansion, and long-term protection against the important human parasite, *T. cruzi*.

**Author Summary**

Over 11 million individuals are infected with *Trypanosoma cruzi,* the causative agent of Chagas disease, which kills >50,000 people annually. Although recent vector control efforts and increased use and effectiveness of chemotherapeutic drugs including benznidazole have reduced infection rates and mortality, a safe, effective vaccine is needed. Vaccination with the *T. cruzi trans*-sialidase (TS) has been used effectively in mice to reduce mortality and chronic disease, however, the establishment of vaccine-induced long-term protective immunity remains elusive. Co-immunization strategies utilizing immune regulators such as interleukin-12 (IL-12) and interleukin-15 (IL-15) can be used to enhance antigen-specific T cell responses and prolong protective immunity. In the present report, we show that genetic vaccination of BALB/c mice with plasmid DNA encoding both TS and IL-15 compared with plasmid DNA encoding TS alone significantly enhanced CD4^+^ and CD8^+^ T cell responses including increased TNF-α, IFN-γ, and IL-2 production, and long-term protection against lethal systemic parasite challenge.

## Introduction

Over 11 million individuals are infected worldwide with *Trypanosoma cruzi*, and over 50,000 deaths are attributed to this protozoan parasite annually [1]. Human infection usually occurs through contact with *T. cruzi* contaminated triatomine excreta, although the parasite also can be transmitted via congenital and parenteral routes or by organ donation [2]. Acute infection, usually associated with only mild and nonspecific symptoms including fever, fatigue, and high parasitemia, often goes undiagnosed. Although high level parasitemia and symptoms resolve after 3–8 weeks, low level tissue parasitism persists for decades, leading to pathologic manifestations of Chagas disease including cardiomyopathy and the mega-syndromes (mega-esophagus and mega-colon) in 30–50% of chronically infected individuals. Chemotherapy with benznidazole and nifurtimox can be highly successful if used within the first several weeks of infection, but rarely leads to cure during chronic infection, and toxic side effects limit their use. Because of the high risk of infection and disease in endemic countries and the lack of well tolerated trypanocidal drugs, a safe and effective vaccine is needed.

Several *T. cruzi* antigens, including *trans*-sialidase (TS), have been used successfully as experimental vaccines to reduce acute *T. cruzi* infection and chronic inflammation in mice [3]–[10]. TS, a member of the largest *T. cruzi* gene family (>1400 genes), is expressed on blood form trypomastigotes (BFT) and metacyclic trypomastigotes (MT), and is also present during early and late stage intracellular infection [11], [12]. *T. cruzi* is unable to synthesize sialic acid, which seems to be required for parasite infectivity. TS cleaves sialic acid from host cell donor molecules and transfers the released sialic acid onto the parasite surface. Vaccines incorporating TS (expressed in adenoviral vectors, in DNA vaccines, or purified recombinant protein mixed with toll-like receptor-9 CpG motifs) induce robust CD4^+^ and CD8^+^ T cell as well as antibody responses in mice, and more importantly, can protect against lethal *T. cruzi* challenge [3], [13]. However, only short term TS vaccine-induced protection has been reported.

Interleukin-15 (IL-15) is one of the so-called ‘homeostatic’ cytokines, which is beneficial for long-term survival of memory T cells. The functions of IL-15 are similar to that of IL-2, although these two cytokines do not have amino acid homology. IL-15 is synthesized by many types of cells including monocytes, macrophages, epithelial, and dendritic cells, but is not expressed by T cells. Central memory T cells (T_cm_) express the receptor for IL-15 (IL-15R), and have the capacities to both extensively proliferate and produce key effector molecules after antigenic restimulation. These central memory T cells are critical for long-term protective immunity. In this work, we describe the enhancement of long term CD8^+^ T cell responses and protective *T. cruzi* immunity in mice (>6 months post vaccination) after vaccination with a combination of DNA vaccines encoding both TS and IL-15.

## Materials and Methods

### Ethics

All animal studies were approved by the Institutional Animal Care and Use Committee (IACUC)/Animal Care Committee (ACC) at Saint Louis University. The University is registered with the USDA as a research facility (43-R-011), is regularly inspected and files an annual report. In addition, under the provisions of the Public Health Service Policy on the Humane Care and Use of Laboratory Animals, revised September 1986, the University has filed the appropriate assurance documents. (Assurance number A-3225-01). The facilities and programs for the use of animals at Saint Louis University are FULLY ACCREDITED by the American Association for the Accreditation of Laboratory Animal Care (AAALAC). The date of our most recent notification was June 18, 2009.

### Parasites and mice

Six to eight week old female BALB/c mice were used throughout these studies (Charles River/NCI). Tulahuén strain *T. cruzi* blood form trypomastigotes (BFT) were prepared by bi-weekly passage through BALB/c mice as previously described [14], [15].

### Optimized IL-15 gene construction

The murine interleukin-15 (IL-15) gene (genbank accession number BC023698) containing the long signal peptide (LSP) was cloned into the Invitrogen pVAX-1 vector (pIL-15LSP), and used as the PCR template for subsequent subcloning strategies. The LSP of IL-15 was replaced with an optimized signal peptide incorporating an efficient Kozak sequence and the 18aa signal peptide derived from *Homo Sapiens* immunoglobulin secretory leader signal as previously described [16]–[18]. This was accomplished using PCR primers 5′-GCCCCCGGATCCGCCGCCACCATGGATTGGACTTGGATCTTATTTTTAGTTGCTGCTGCTACTAGAGTTCATTCTAACTGGATAGATGTAAGATAT-3′ and 5′-GCCCCCGAATTCTCAGGACGTGTTGATGAACATTTG-3′. The resulting PCR product was subcloned into pVAX-1 using BamH1 and EcoR1 restriction sites (pIL-15opt, later referred to as pIL-15). Plasmids (pVAX, pIL-15LSP, and pIL-15opt) were propagated in DH5α strain *E. coli* and purified using QIAGEN plasmid purification kits (Valencia, CA).

### Measurement of IL-15 expression in COS-7 cells

COS-7 cells (ATCC, Manassas, VA) were transfected with pVAX, pIL-15LSP, or pIL-15opt using Lipofectamine 2000 transfection reagent (Invitrogen) as recommended by the manufacturer. After 48 hours, cell lysates were prepared for use in IL-15-specific western blots and IL-15 bioassays. Western blots were performed using biotinylated goat anti-mouse IL-15 and streptavidin-HRP, followed by development with Luminol chemiluminescent substrate. In addition, lysates from transfected COS-7 cells were added to 96 well flat bottom plates containing 5×10^3^ CTLL-2 cells (IL-15 sensitive cell line, ATCC) and incubated at 37°C for 24 hours prior to the addition of 0.5 µCi [^3^H] thymidine. After 24 hours, cells were harvested onto glass fiber filtermats using a Tomtec Mach-IIIM automated cell harvester, Ultima Gold F scintillation fluid added, and radioactive incorporation measured using a Wallac Microbeta 1450 scintillation counter [3]. A standard curve of mIL-15 was generated using purified rmIL-15, and employed to determine levels of functional IL-15 present in transfected COS-7 cell lysates (reported in ng/ml).

### Plasmid DNA vaccination of mice

Negative control (pcDNA), *Trans*-sialidase (pTS; genbank D50685, GI 840707 described in [19]) and IL-15 optimized (pIL-15) plasmids were prepared using QIAGEN EndoFree Plasmid Giga kits as per manufacturer recommendations. Plasmids were suspended at a concentration of 2 mg/ml in sterile endotoxin free PBS. Endotoxin levels were <1 endotoxin unit/ml as determined by LAL assay (Associates of Cape Cod, East Falmouth, MA). Mice were anesthetized intraperitoneally with ketamine (60 mg/kg) and xylazine (5 mg/kg), then vaccinated i.m. (tibialis anterior, 50 µl per limb) three times at two week intervals with a total of 200 µg plasmid DNA (100 µg pcDNA + 100 µg pIL-15, 100 µg pcDNA + 100 µg pTS, or 100 µg pIL-15 + 100 µg pTS). Representative mice were harvested 1, 3, or 6 months after their final immunization to assess vaccine-induced immune responses. Other mice (N = 5–10 per group) were challenged with 5,000 BFT subcutaneously and survival followed for > 2 months.

### Quantitation of IFN-γ secreting cells by ELISPOT

One to six months after the third and final vaccination, spleen cells from representative vaccinated mice were stimulated in IFN-γ ELISPOT assays. Spleen cells (3–5×10^5^ per well) plus antigen presenting cells {1×10^5^ A20J cells alone (ATCC), A20J cells pulsed with 2.5 µg/ml of the immunogenic CD8^+^ H2K^d^ restricted TS peptide IYNVGQVSI (TSaa359-367;ResGen/Invitrogen) [3], [20], or A20J transfected with the TS gene [21]} were cultured in anti-IFN-γ coated BD Biosciences, San Jose, CA) and blocked nitrocellulose bottom ELISPOT plates [using T-cell^+^ media: 10% FBS (Thermo Scientific Hyclone, Logan, UT), 50% EHAA (Invitrogen, Carlsbad, CA), 37% RPMI (Invitrogen) 50 U/ml penicillin + 50 µg/ml streptomycin (Lonza Inc., Allendale, NJ), 2 mM L-Glutamine (Lonza), 50 µM 2-mercaptoethanol (Sigma, St. Louis, MO), and 50 µg/ml gentamycin (Lonza)]. After overnight stimulation, wells were subsequently incubated with 1) biotinylated-anti-mouse IFN-γ BD Biosciences), 2) streptavidin-HRP (Jackson Immunoresearch Laboratories, West Grove, PA), 3) and AEC substrate (Sigma). Images of developed ELISPOT plates were captured using a CTL Analyzer and spots counted using Immunospot Software v3.2 (CTL, Shaker Heights, OH). Data are represented as the number of IFN-γ spot forming cells (SFC) per million total spleen cells (TSC) or SFC per million CD8^+^ T cells.

### TS-specific IgG ELISA

TS-specific serum IgG responses were measured by ELISA in samples collected from individual mice 1, 3, and 6 months post-immunization as previously described [3]. Endpoint titers were determined using Unitwin Opticalc software (PhPlate AB, Stockholm Sweden) based upon transformed O.D. and dilution curves using a 0.2 O.D. cutoff.

### Measurement of cytokine producing cells by intracellular cytokine staining (ICS) and flow cytometry

Spleen cells (2×10^6^/ml) from mice vaccinated 6 months prior were stimulated with 1×10^5^/ml A20J cells or A20J transfected with TS (A20-TS) in round bottom culture tubes. After overnight culture, monensin and brefeldin-A were added as recommended by the manufacturer (GolgiPlug and GolgiStop, respectively, BD Biosciences). In order to study polyclonal T cell responses, cells were rested overnight prior to addition of phorbol-12-myristate 13-acetate (PMA, 10 ng/ml), ionomycin (500 ng/ml), monensin, and brefeldin-A for the final 3 hours of culture. Cells were then washed with PBS, stained using LIVE/DEAD fixable Aqua Stain cell reagent, (Invitrogen), Fc receptors blocked by incubation with anti-CD16/32 (15 minutes, 4^o^C), then surface stained at 4^o^C for 30 minutes using CD3-FITC, CD8-PerCP, CD4-Pacific Blue, and TS^(IYNVGQVSI)^/H2K^d^-tetramer-APC (all antibodies were purchased from BD Biosciences unless noted otherwise, and the IYNVGQVSI-specific APC tetramer reagent was provided through the NIH Tetramer Core Facility, Atlanta, GA). BD Cytoperm/Cytofix was used to fix and permeablize the cells as per manufacturer suggestions prior to staining with IL-2-PE, IFN-γ-Alexa700 and TNF-α-PE-Cy7 (30 minutes at 4^o^C). Cells were then washed and resuspended in permeabilization buffer prior to acquisition on a LSR-II flow cytometer (BD). Data sets were analyzed using FLOWJO Flow Cytometry Analysis v7 software (Tree Star, Inc., Ashland, OR).

### Analysis of TS^(IYNVGQVSI)^/H2K^d^ tetramer positive cells

Six months following immunization, spleen cells were stained directly ex vivo using the surface staining procedure described above. Additionally, spleen cells were stained with CFSE (Vybrant CFDA SE cell tracer, Invitrogen), washed 3 times, and cultured (2×10^6^/ml) with irradiated (12,500 rads) A20J or A20-TS cells (1×10^5^) for 6 days at 37^o^C using the same media as described above (T-cell^+^). Cells were then washed and stained for CD3, CD8, CD4, and TS^(IYNVGQVSI)^/H2K^d^ tetramer binding prior to flow cytometric analysis as described above. Absolute numbers (AN) of TS^(IYNVGQVSI)^/H2K^d^ tetramer positive cells present in expanded cultures were determined as follows: total cell yield after expansion as determined by trypan blue cell count × (# of TS^(IYNVGQVSI)^/H2K^d^ tetramer positive events ÷ number of live events collected). These ANs were used to calculate the antigen-specific AN index (AN Index = absolute number of TS^(IYNVGQVSI)^/H2K^d^ tetramer positive cells after A20-TS expansion ÷ absolute number of TS^(IYNVGQVSI)^/H2Kd-tetramer positive cells present after NC A20 expansion).

### Adoptive transfer studies

Adenoviral vectors (type 5) encoding TS (Adeno-TS) were constructed as described previously [13]. HEK293A cells were grown at 37^o^C to 80% confluency in T150 flasks using 10% FBS in DMEM supplemented with penicillin, streptomycin, and L-glutamine. Flasks were inoculated with recombinant adenovirus diluted in 5 ml of DMEM alone. After 2 hours, complete media was added and infected cells cultured until cytopathic effect observed (usually 48 hours after infection). Cells were manually detached and pooled (20 flasks/prep), pelleted by centrifugation, supernatants discarded, and cells resuspended in 20 ml of complete DMEM. Cells were freeze-thawed 3 times, sonicated for 3 minutes (3 times), and centrifuged at 17,000×*g* for 10 minutes at 4^o^C. CsCl was added to 0.51 g/ml, mixed for 20 minutes at room temperature, and centrifuged at 110,000×*g* for 24 hours at 4^o^C. Adenovirus bands were collected, dialyzed into 20 mM Tris, pH 8.0 (10,000 MWCO, 3 exchanges), and glycerol added (final concentration 10%) prior to storage at −80^o^C. Standard TCID_50_ assays using HEK293A cells were used to determine adenovirus stock concentrations.

BALB/c mice were vaccinated i.m. on days 0 and 14 with 100 µg of pTS alone, and boosted on days 42 and 56 with 1×10^8^ pfu of Adeno-TS s.c. Two months later, spleen cells were harvested from TS prime-boost vaccinated mice and CD8^+^ T cell fractions prepared using positive-selection standard magnetic bead separation techniques (Miltenyi Biotec, Bergisch Gladbach, Germany ; typical CD8^+^ fraction purities exceeded 98%). Functionality of CD8^+^ T cells was confirmed by IFN-γ ELISPOT as described above. Purified CD8^+^ T cells were injected i.p. into naïve BALB/c recipient mice (2.6×10^7^ per mouse), and mice challenged 24 hours later with 5,000 BFT s.c.

### Statistical analyses

Data sets were analyzed using Statistica v. 6.0 (Statsoft, Inc., Tulsa, OK). Tests performed include Mann Whitney U tests, Fisher's exact tests, and Wilcoxon Matched pairs tests.

## Results

### Construction and functional testing of the IL-15 plasmid DNA

**Figure 1:**
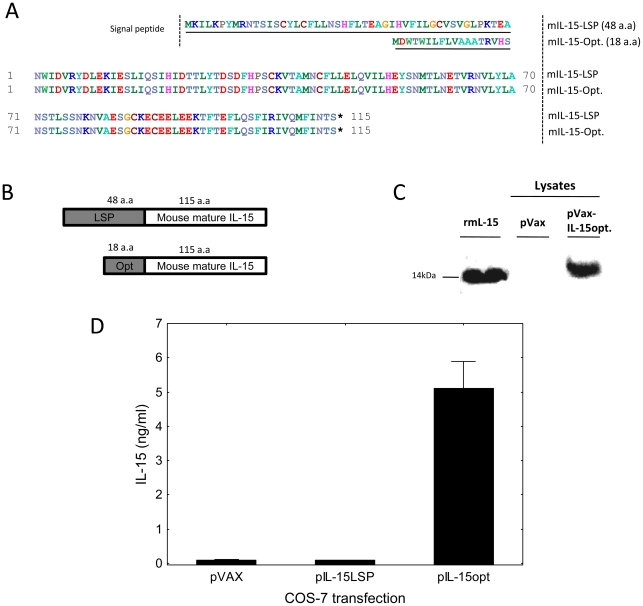
Construction and evaluation of IL-15 expression plasmids. Shown in panels A and B are amino acid alignments and mature protein schematics of the murine IL-15 genes containing the long signal peptide (LSP) and optimized signal sequence (opt). These proteins were cloned into the eukaryotic expression plasmid pVAX and resulting plasmids transfected into COS-7 cells. After 48 hours, lysates from pVAX and pIL-15opt transfected COS-7 cells were prepared and analyzed by western blot along with recombinant murine IL-15 (rmIL-15, panel C). COS-7 cells transfected with pIL-15opt resulted in high level expression of murine IL-15. IL-2/IL-15 dependent cells (CTLL-2) were cultured in 96 well plates with transfected COS-7 lysates in order to determine the functionality of the cloned IL-15 constructs. Purified recombinant IL-15 was utilized as a positive control and used to prepare a standard curve. After 24 hours of culture, ^3^H-thymidine was added and one day later plates were harvested, scintillated, and radioactive incorporation measured. COS-7 cells transfected with pIL-15opt express functionally active IL-15 as determined using this CTLL-2 bioassay (D).

The murine IL-15 gene containing the long signal peptide (LSP) was cloned into the mammalian expression plasmid pVAX as described in Materials and Methods. We inserted an optimized signal sequence for IL-15 which includes a stronger Kozak sequence, removal of upstream inhibitory AUG sequences, and changed the LSP to the *Homo Sapiens* immunoglobulin secretory leader signal as previously described [16]–[18]. The complete amino acid sequences and protein schematics of the IL-15 genes are shown in Figs. 1A and 1B. Plasmid DNA encoding murine IL-15 sequences with or without the upstream optimization sequence (pIL-15LSP and pIL-15opt, respectively) were transiently transfected into COS-7 cells, and after 48 hours cell lysates were prepared and analyzed for IL-15 expression using IL-15-specific western blots and IL-15-sensitive cells (CTLL-2 bioassays). COS-7 cells transfected with the optimized IL-15 plasmid but not with the pVAX alone expressed IL-15 as shown by western blot (Fig. 1C). The functional activity of the COS-7 synthesized IL-15 is shown in Fig. 1D. These results demonstrate that the cloned optimized murine IL-15 gene can be highly expressed and has potent functional activity in mammalian cells (pIL-15opt referred to as pIL-15 hereafter).

### Immunization with trans-sialidase DNA induces robust TS-specific IFN-γ responses and protection against lethal T. cruzi challenge given 1 month post-vaccination

**Figure 2:**
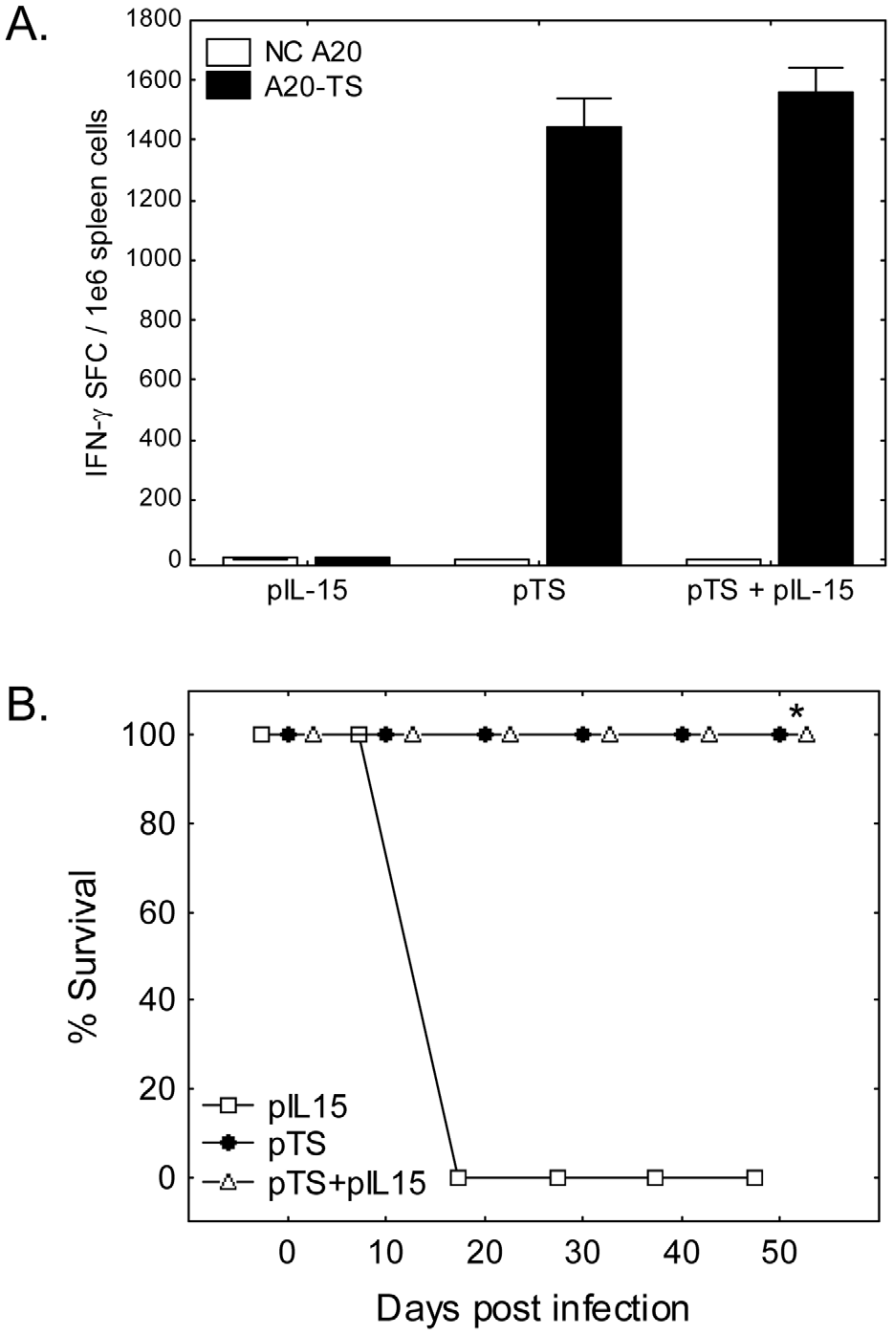
T cell responses and protective immunity at 30 days post pTS ± IL-15 vaccination. BALB/c mice were vaccinated i.m. 3 times, 2 weeks apart, with plasmids encoding TS (pTS) and/or IL-15 (pIL-15). Thirty days after the final vaccination, spleen cells from representative mice were incubated for 16–20 hours in the presence of negative control antigen presenting cells (NC A20), or antigen presenting cells stably transfected with full length TS (A20-TS) in IFN-γ ELISPOT assays. In addition, 5 mice per group were challenged with 5,000 BFT s.c. and survival monitored for > 2 months. Mice vaccinated with pTS (regardless of inclusion of pIL-15) produced high numbers of TS-specific IFN-γ secreting cells (A, pooled cells from 2 mice per group). Additionally, 100% of mice vaccinated with pTS or pTS+pIL-15 survived lethal systemic *T. cruzi* challenge, whereas all mice vaccinated with control plasmids succumbed to infection (B, * P<0.05 by Fisher's exact tests in comparison to pIL-15 alone group). Data representative of 3 experiments.

We have previously shown that immunization using plasmid DNA encoding TS or recombinant TS protein (rTS) mixed with CpG adjuvant induces strong TS-specific IFN-γ responses and protection against lethal systemic *T. cruzi* challenge [3]. In order to demonstrate the effectiveness of vaccination with IL-15-adjuvanted TS, BALB/c mice were immunized with pcDNA + pIL-15, pcDNA + pTS, or pIL-15 + pTS as described above. Thirty days after the final vaccination spleen cells from representative mice were stimulated with antigen presenting cells permanently transfected (or not) with DNA encoding TS in IFN-γ ELISPOT assays {A20 and A20-TS, [21]}. Regardless of co-immunization with pcDNA or pIL-15, all mice vaccinated with pTS generated similar numbers of TS-specific IFN-γ secreting T cells detectable at the 1 month post-vaccination time point (Fig. 2A). In addition, spleen cells from all groups of mice receiving pTS vaccination contained similar numbers of IYNVGQVSI-specific CD8^+^ T cells as indicated by IFN-γ ELISPOT (1.1±1.1, 51.1±7.3, and 54.4±4.8 TS^IYNVGQVSI^-specific IFN-γ spot forming cells per million spleen cells in pIL-15 control, pTS alone, and pTS + pIL-15 vaccinated mice, respectively). We were unable to detect differences in TS-specific IgG responses in sera from pTS and pTS + pIL-15 vaccinated mice (endpoint titers of >90,000 in pTS alone and pTS + pIL-15 vaccinated mice, compared to <100 detected in samples from pIL-15 alone vaccinated mice). In order to study protective immunity induced by the different vaccination strategies, we challenged 5 mice per group with a lethal dose of *T. cruzi* BFT (5,000 BFT s.c.) 30 days following the final immunization, and followed survival for >2 months. All mice vaccinated with pTS (with or without pIL-15 co-immunization) survived lethal *T. cruzi* challenge, whereas all mice vaccinated with pIL-15 alone died (Fig. 2B, P<0.05 comparing pIL-15 alone group to pTS alone and pTS + pIL-15 vaccinated groups).

### Intermediate term immune responses and systemic protection by pTS & pIL-15 vaccination

**Figure 3:**
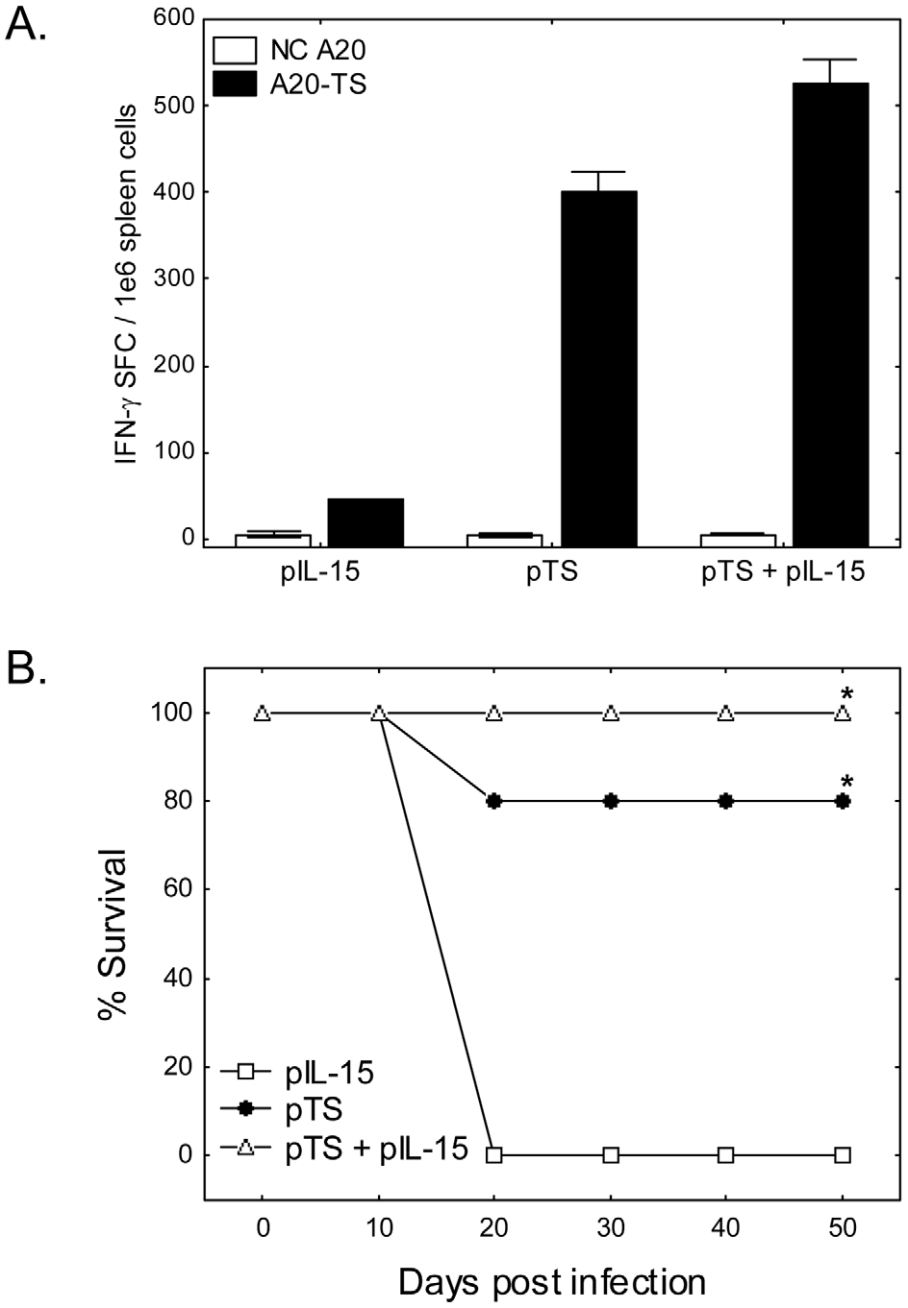
T cell responses and protective immunity at 90 days post pTS ± IL-15 vaccination. Female BALB/c mice were vaccinated i.m. 3 times, 2 weeks apart, with plasmid DNAs encoding TS (pTS) and/or IL-15 (pIL-15). Three months after the third and final vaccination, spleen cells from representative mice were incubated for 16-20 hours in IFN-γ ELISPOT assays in the presence of negative control antigen presenting cells (A20), or antigen presenting cells stably transfected with full length TS (A20-TS). In addition, 5 mice per group were challenged with 5,000 BFT s.c. and survival monitored for > 2 months. Mice vaccinated with pTS (regardless of inclusion of pIL-15) produced high numbers of TS-specific IFN-γ secreting cells detected 3 months following immunization (A, pooled cells from 2 mice per group). Additionally, 4 of 5 mice vaccinated with pTS and all 5 of the mice vaccinated with pTS+pIL-15 survived systemic lethal *T. cruzi* challenge (B, * P<0.05 by Fisher's exact tests in comparison to pIL-15 alone group). Data representative of 3 experiments.

In mice three months after vaccination with plasmids encoding TS and/or IL-15, we analyzed vaccine-induced TS-specific T cell responses utilizing IFN-γ ELISPOT assays and induction of protective immunity against lethal *T. cruzi* challenge. As shown in Fig. 3A., vaccination of mice with pTS alone or pTS + pIL-15 elicited strong TS-specific T cell responses as detected 3 months after vaccination. However, there was a trend for higher frequencies of TS-specific IFN-γ producing cells after pTS co-immunization with pIL-15 (pTS + pIL-15>pTS alone by >20%). There were no significant differences in TS-specific serum IgG responses present in serum samples obtained from pTS alone and pTS + pIL-15 vaccinated mice (endpoint titers of >90,000 in pTS alone and pTS + pIL-15 samples compared to <100 in pIL-15 alone vaccinated mice). Immunization of mice with pTS alone and pTS + pIL-15 resulted in significantly enhanced survival after challenge compared with pIL-15 immunization alone (Fig 3B, P<0.05). Additionally, although not statistically significant, 4 of 5 mice vaccinated with pTS alone survived lethal *T. cruzi* challenge compared with 5 of 5 mice vaccinated with pTS + pIL-15.

### Long-term Protective Immunity induced by pTS & pIL-15 co-immunization

**Figure 4:**
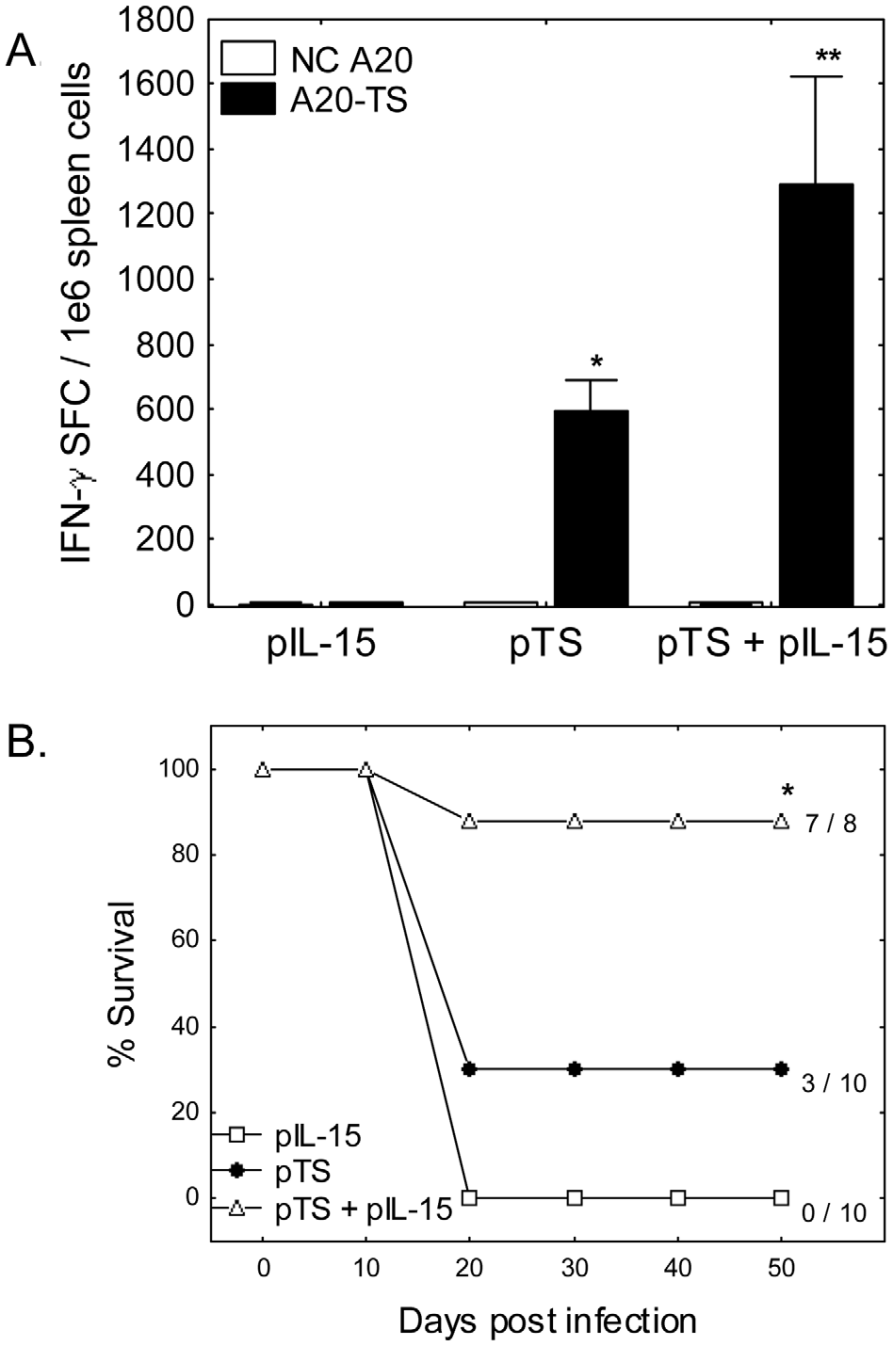
T cell responses and protective immunity at 6 months post pTS ± IL-15 vaccination. BALB/c mice were vaccinated i.m. 3 times, 2 weeks apart, with plasmids encoding TS (pTS) and/or IL-15 (pIL-15). (A) Six months after the final vaccination, spleen cells from representative mice (4 mice per group) were incubated for 16-20 hours in IFN-γ ELISPOT assays in the presence of negative control antigen presenting cells (NC A20), or antigen presenting cells stably transfected with full length TS (A20-TS). Mice vaccinated with pTS + pIL-15 developed significantly increased numbers of TS-specific IFN-γ secreting cells compared with mice vaccinated with pTS alone (A*P<0.05 compared with the pIL-15 alone group, **P<0.05 compared with either the pIL-15 alone or pTS alone groups by Mann Whitney U tests). (B) In addition, 8–10 mice per group were challenged with 5,000 BFT s.c. and survival monitored for >2 months. Only 3 of 10 mice vaccinated with pTS alone, and none of 10 mice vaccinated with pIL-15 alone, survived lethal *T. cruzi* challenges (differences not significant). In contrast, 7 of 8 mice vaccinated with pTS+pIL-15 survived *T. cruzi* BFT challenge (P<0.05 comparing pTS+pIL-15 to pTS alone, and comparing pTS+pIL-15 to pIL-15 alone). Data representative of 2 experiments.

IL-15 is thought to enhance long term survival of memory T cells, thus we explored long term T cell function in pTS alone versus pTS + pIL-15 vaccinated mice. Six months following the final immunization, we detected nearly twice as many TS-specific IFN-γ producing T cells in spleens from mice co-immunized with pTS + pIL-15 compared with spleen cells obtained from mice vaccinated with pTS alone (Fig. 4A, P<0.05 by Mann Whitney U test comparing pTS alone to pIL-15 + pTS groups.). Vaccination with pTS alone and pTS + pIL-15 resulted in potent TS-specific serum IgG responses detected 6 months post immunization [endpoint titers in serum samples from pTS alone and pTS+pIL-15 immunized mice were >15,000 compared to <100 in pIL-15 controls (P<0.05 comparing pTS alone and pTS+pIL-15 groups to pIL-15 control group)]. In addition, protective immunity induced by pTS vaccination alone was significantly reduced 6 months after vaccination, as demonstrated in Fig. 4B. Only 3 of 10 mice vaccinated with pTS alone, and none of 10 mice vaccinated with pIL-15 alone, survived lethal *T. cruzi* challenges (differences not significant). In contrast, 7 of 8 mice vaccinated with pTS + pIL-15 survived *T. cruzi* BFT challenge (P<0.05 comparing pTS + pIL-15 to pTS alone, and comparing pTS + pIL-15 to pIL-15 alone). Thus, addition of IL-15 to the TS vaccination regimen significantly enhances long term survival against lethal parasite challenge.

### IL-15 induces long-term CD4^+^ and CD8^+^ T cell cytokine production potential

After demonstrating that co-administration of pIL-15 with pTS greatly enhances long-term protection against *T. cruzi* challenge, we further dissected T cell responses induced by vaccination with pTS alone compared with pTS + pIL-15. In order to study T cell compartment cytokine production potentials, we stimulated spleen cells from vaccinated mice (6 months after the 3^rd^ and final vaccination) with PMA + ionomycin, then analyzed the overall cytokine production in T cells using intracellular flow cytometry. As seen in Table 1, in general, pTS vaccination alone did not significantly alter CD4^+^ or CD8^+^ T cell profiles in comparison with control pIL-15 vaccinated mice in terms of IL-2, IFN-γ, or TNF-α expression. However, vaccination with pTS + pIL-15 resulted in significantly increased frequencies of all cytokine parameters analyzed in both CD4^+^ and CD8^+^ T cell compartments (IL-2, IFN-γ, and TNF-α, P<0.05 by Mann Whitney comparing pTS+pIL-15 to pTS alone and pIL-15 alone). In addition, we detected significantly increased frequencies of multi-parameter CD4^+^ and CD8^+^ T cells which expressed IL-2 + IFN-γ as well as TNF-α + IFN-γ (P<0.05 by Mann Whitney comparing pTS+pIL-15 to pTS alone and pIL-15 alone).

**Table 1:** Increased Type 1 phenotype of CD4^+^ and CD8^+^ T cells 6 months after administration of pTS + pIL-15.

	pIL-15	pTS	pTS + pIL-15
CD4 gated, IL2^+^	0.66±0.22	0.99±0.24	6.83±0.51^‡^
CD4 gated, IFNγ^+^	0.54±0.16	0.52±0.13	2.39±0.33^‡^
CD4 gated, TNFα^+^	11.64±3.27	9.56±2.10	50.64±1.99^‡^
CD4 gated, IL2^+^IFNγ^+^	0.09±0.03	0.15±0.03	0.74±0.07^‡^
CD4 gated, TNFα^+^IFNγ^+^	0.39±0.11	0.43±0.10	1.85±0.25^‡^
CD8 gated, IL2^+^	0.04±0.00	0.09±0.02	0.53±0.09^‡^
CD8 gated, IFNγ^+^	0.19±0.06	0.37±0.04	1.45±0.11^‡^
CD8 gated, TNFα^+^	9.62±2.53	8.63±1.62	44.82±2.10^‡^
CD8 gated, IL2^+^IFNγ^+^	0.01±0.01	0.03±0.00	0.13±0.03^‡^
CD8 gated, TNFα^+^IFNγ^+^	0.16±0.04	0.32±0.04†	1.15±0.07^‡^

Shown are frequencies of cytokine producing cells 6 months following vaccination after PMA+ionomycin stimulation (mean±SE from N = 4–5 per group).

**†:** P<0.05 compared to pIL-15 alone vaccine group; ^‡^ P<0.05 compared to pIL-15 and pTS vaccine groups.

### IL-15 increases TS-specific, IFN-γ producing CD8^+^ T cell numbers and proliferative capacity

**Figure 5:**
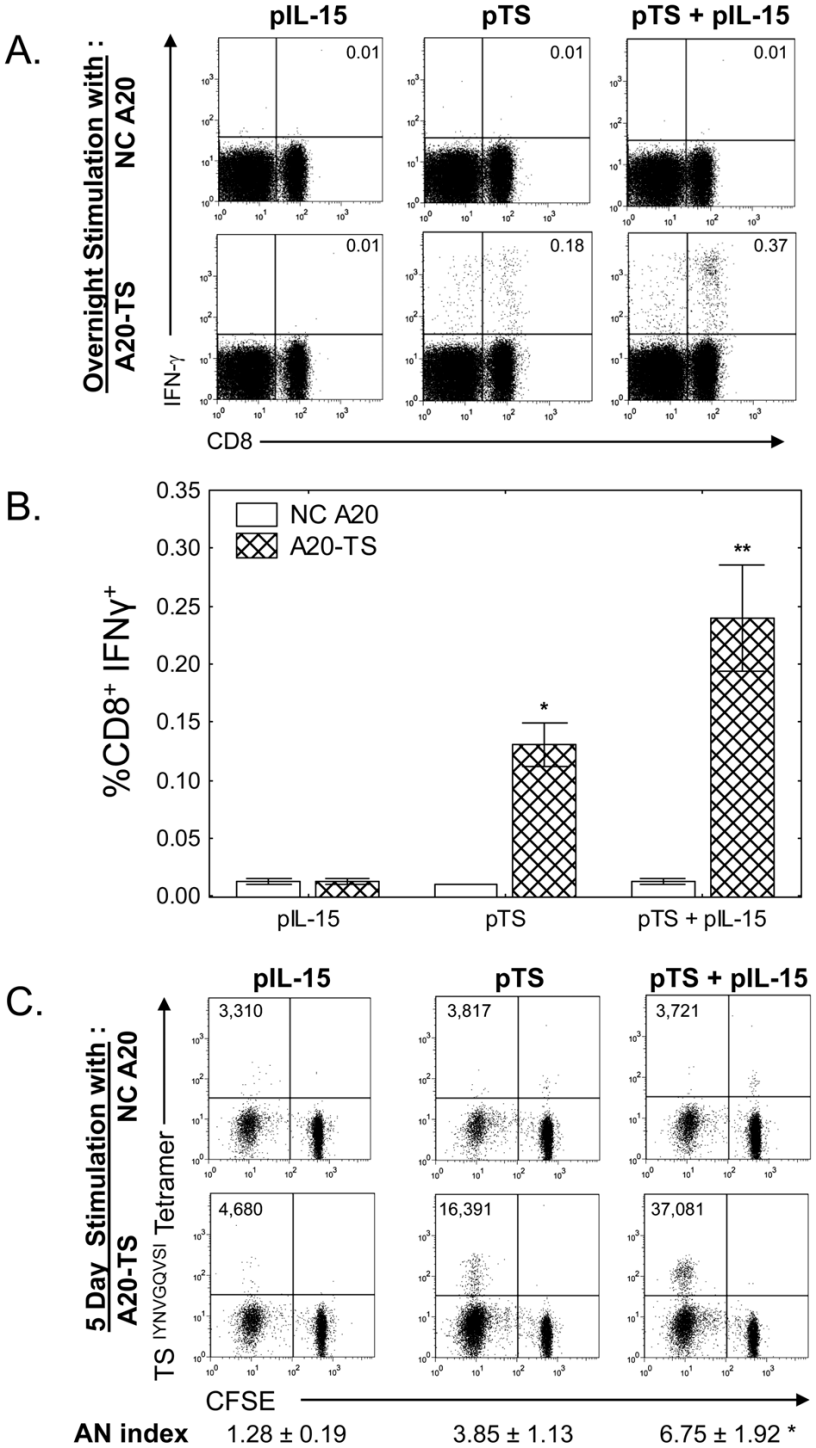
Persistent increases in CD8^+^ T cell frequencies and responsiveness after co-administration of pTS + pIL-15. BALB/c mice were vaccinated three times, 2 weeks apart with pIL-15, pTS, or pIL-15+pTS. Six months later, spleen cells (N = 4/group) were cultured overnight with negative control (NC A20) or TS-expressing APC (A20-TS), then ICS performed for quantitation of TS-specific IFN-γ producing CD8^+^ T cells (A and B, shown are frequencies of CD3^+^ T cells). Spleen cells from mice vaccinated with pTS + pIL-15 exhibited much higher frequencies of TS-specific IFN-γ expressing CD8^+^ T cells compared with spleen cells from mice vaccinated with pTS alone (representative results shown in A; cumulative data from 4 individual mice per group in B). In order to study expansion capacities of TS-specific CD8^+^ T cells, spleen cells from mice obtained 6 months post vaccination (N = 4 per group) were labeled with CFSE and co-cultured with irradiated negative control A20 or A20-TS APCs. On day 5, T cells were stained for T cell surface markers including a tetramer reagent specific for TCR reactive with an immunodominant H2K^d^-restricted epitope (TSKd1) and studied by flow cytometry (gated on CD8^+^ T cells). Shown in Panel C are the absolute numbers of TSKd1-specific T cells (in upper left of FACS plots), and the expansion indices of TSKd1-specific T cells detected in these assays (AN index = absolute number of TSKd1-specific cells after A20-TS expansion ÷ absolute number of TSKd1-specific cells after NC A20 expansion.) Vaccination with pTS + pIL-15 resulted in significant enhancement of TSKd1–specific CD8^+^ T cell responses capable of lymphoproliferative expansion. (* P<0.05 in comparison with pIL-15 alone group, ** P<0.05 in comparison with pIL-15 alone and pTS alone vaccine groups by Mann Whitney U tests.) Data representative of 2 experiments.

Both CD4^+^ and CD8^+^ T cells from mice vaccinated with pTS + pIL-15 exhibited more potential for robust cytokine production following polyclonal stimulation with PMA + ionomycin than cells obtained from mice vaccinated with pTS alone. In order to determine the frequencies of CD4^+^ and CD8^+^ IFN-γ producing TS-specific T cells at the late time point in which we observed differential protective capacity against lethal *T. cruzi* challenge, we cultured total spleen cells from vaccinated mice overnight with APC transfected (or not) with full length TS, then performed intracellular cytokine staining as detailed above. We detected significant increases in frequencies of CD4^+^ T cells producing IFN-γ, TNF-α, and IL-2 upon A20-TS stimulation in mice vaccinated with either pTS or pTS + pIL-15 compared to mice vaccinated with control pIL-15 (P<0.05 by Wilcoxon matched pair and Mann-Whitney U tests), however, there were no noticeable differences measured between pTS alone and pTS + pIL-15 groups (Fig. S1). Similar statistically significant increases in TS-specific IFN-γ, IL-2, and TNF-α producing cells amongst CD8^+^ T cells were observed in pTS and pTS + pIL-15 vaccinated groups compared with the pIL-15 control vaccinated group (Figs. 5A, B, and Fig. S2, P<0.05 by Wilcoxon matched pair and Mann-Whitney U tests). As shown in Figs. 5A and B, significantly more TS-specific IFN-γ^+^ splenic CD8^+^ T cells were detected in mice vaccinated 6 months prior with pTS + pIL-15 compared with mice vaccinated with pTS alone (Fig 5A, FACS plots from representative mice; Fig 5B, cumulative data from N = 4 mice per group, P<0.05 by Mann Whitney U test).

Vaccination of BALB/c mice with pTS has previously been shown to induce potent CD8^+^ T cell responses against the immunodominant TS H2K^d^-restricted peptide IYNVGQVSI. We anticipated that we would observe higher frequencies of TS^(IYNVGQVSI)^-specific T cells in pTS + pIL-15 vaccinated mice compared with pTS alone vaccinated mice. However, even though we observed statistically significant increases in frequencies of CD8^+^ TS^(IYNVGQVSI)^/H2K^d^ tetramer-specific T cells 6 months post vaccination in both pTS and pTS + pIL-15 immunized mice compared with pIL-15 control vaccinated mice, we could not detect differences between the pTS and pTS + pIL-15 vaccinated groups (0.088%±0.025 and 0.088%±0.021 in pTS and pTS + pIL-15 groups, respectively, compared with 0.035%±0.005 in pIL-15 control mice as determined by tetramer staining, P<0.05 by Mann Whitney). In order to analyze the proliferative capacity of these TS^(IYNVGQVSI)^-specific CD8^+^ T cells after exposure to antigen, we labeled spleen cells from vaccinated mice with CFSE and stimulated these cells with irradiated APC stably transfected (or not) with TS for 5 days prior to tetramer staining. As shown in Fig. 5C, TS^(IYNVGQVSI)^ -specific CD8^+^ spleen cells from pTS + pIL-15 vaccinated animals expanded to greater frequencies and absolute numbers compared with cells obtained from pTS alone vaccinated mice (P<0.05 comparing pTS+pIL-15 and pIL-15 groups).

### Trans-sialidase-specific CD8^+^ T cells alone can protect against T. cruzi challenge

**Figure 6:**
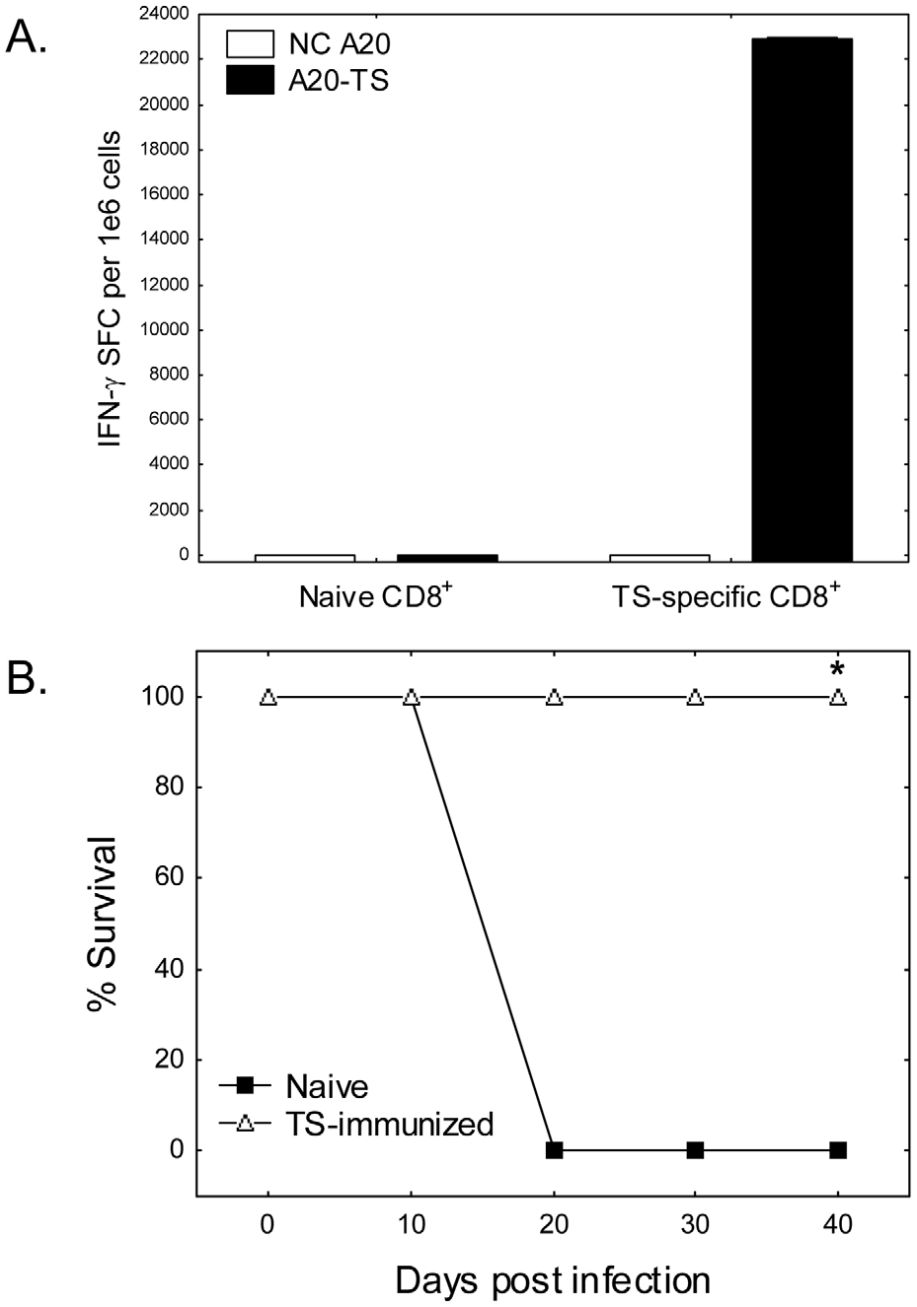
Vaccine-induced TS-specific CD8^+^ T cells mediate protection against lethal systemic *T. cruzi* challenge. BALB/c mice were vaccinated with 100 µg of pTS alone i.m. on days 0 and 14 followed by 1×10^8^ pfu Adeno-TS s.c. on days 42 and 56 (A and B, data representative of 4 experiments). Two months later, immunomagnetically purified splenic CD8^+^ T cells from vaccinated mice were stimulated with negative control or TS-expressing APC (A20 and A20-TS) in IFN-γ ELISPOT assays (A). Mice vaccinated with pTS followed by Adeno-TS developed greatly increased CD8^+^ TS-specific IFN-γ responses (>20,000 SFC/million). CD8^+^ T cells obtained from negative control or TS prime-boost vaccinated mice were injected i.p. into naïve BALB/c recipient mice (2.6×10^7^ per mouse). These adoptively transferred mice were challenged the following day with 5,000 *T. cruzi* BFT s.c. (N = 4 mice per group; B). Mice adoptively transferred with negative control T cells succumbed rapidly to *T. cruzi* infection. However, all mice which received TS-specific CD8^+^ T cell transfers survived lethal *T. cruzi* challenge (* P<0.05, Fisher's exact test).

As total TS-specific IFN-γ T cell responses were found to be greatly increased after addition of pIL-15 to the pTS vaccination regimen 6 months post-vaccination, and these increased responses were associated with increased protection, we next sought to investigate whether pTS-induced CD8^+^ T cells alone could transfer protection against lethal *T. cruzi* challenge. The frequencies of CD8^+^ T cells present in mice vaccinated with pTS DNA with or without pIL-15 have not been sufficient to allow practical adoptive transfer experiments to be done. We have recently found that a strategy of boosting pTS responses with TS-expressing adenovirus vaccinations results in up to 10-20 fold more TS-specific CD8^+^ T cells. Therefore, we vaccinated BALB/c mice with 2 doses of pTS DNA followed 1 month later with 2 doses of Adeno-TS to obtain sufficient numbers of TS-specific CD8^+^ T cells for adoptive transfer experiments. As shown in Fig. 6A, mice vaccinated with pTS followed by Adeno-TS developed massive CD8^+^ TS-specific IFN-γ responses (>22,000 IFN-γ spot forming cells per million CD8^+^ T cells). We adoptively transferred 2.6×10^7^ CD8^+^ T cells obtained from negative control or TS prime/boost vaccinated mice into naïve BALB/c recipient mice followed by challenge of these mice the following day with a lethal dose of *T. cruzi* (5,000 BFT s.c.). As seen in Fig. 6B, mice adoptively transferred with negative control T cells succumbed rapidly to *T. cruzi* infection (100% mortality within 3 weeks). However, all of the mice which received TS-specific CD8^+^ T cell transfers survived lethal *T. cruzi* challenge (P < 0.05, Fisher's exact test). Thus, vaccine-induced TS-specific CD8^+^ T cells alone can protect against lethal systemic *T. cruzi* challenge, and the increases in TS-specific CD8^+^ T cell responses seen in mice vaccinated with pTS + pIL-15 could be sufficient to explain the mechanism for prolonged enhanced protection.

## Discussion

The addition of IL-15 adjuvants to vaccine regimens has previously been shown to enhance protective immunity against a variety of pathogens, including influenza, *Toxoplasma gondii*, and Herpes simplex virus (HSV) [22]–[24]. We have demonstrated here that the addition of a plasmid DNA expressing murine IL-15 (pIL-15) to a plasmid DNA vaccine encoding the *T. cruzi trans*-sialidase (pTS) leads to significant enhancement of long-term protective immunity against this complex intracellular protozoan pathogen. The combination of pIL-15 and pTS induced significantly higher levels CD4^+^ Th1 cells and TS-specific IFN-γ producing CD8^+^ T cells which displayed enhanced proliferative capacity after antigenic restimulation compared with the levels of these responses induced by vaccination with pTS alone. More importantly, these TS-specific immune responses enhanced by IL-15 co-administration led to significant increases in protective immunity 6 months post-vaccination.

IL-15 is a member of a family of common γ chain (γc)-dependent cytokines including IL-2, IL-4, IL-7, IL-9, IL-15 and IL-21, which require expression of the γc receptor component for functional cytokine signaling. The IL-15 and IL-2 receptor complexes also share CD122 (IL-2Rβ/IL-15Rβ) resulting in very similar in vitro effects of these 2 cytokines on the growth of NK cells and different subsets of T cells. However, IL-15 signaling is unique among the γc cytokines involving a plasma membrane/endosomal recycling of the complex between IL-15 and its unique IL-15Rα receptor component which results in the prolonged capacity for IL-15 to signal in *trans* to cells expressing the γc/IL-15Rβ complex on their surface [25]. In contrast, efficient signaling by soluble IL-2 requires binding to the high affinity IL-2R complex (including CD25/γc/IL-2Rβ) followed by receptor/cytokine internalization and degradation of the IL-2 ligand. Hence, IL-15 signaling is prolonged and dependent on cell-to-cell contact while IL-2 signaling is more transient and requires active IL-2 secretion by activated cells. These biological differences and the distinct expression profiles of CD25 and IL-15Rα likely explain the opposing effects of IL-15 and IL-2 identified in vivo [26]. Deficiency of IL-15 leads to a profound depletion of NK cells, NKT cells, intestinal intraepithelial lymphocytes and CD8^+^ memory T cells, whereas deficiency of IL-2 leads to lymphoproliferative and autoimmune disease. In addition, IL-15 has overlapping functions with IL-7 important for the survival, maintenance and homeostatic proliferation of both CD4^+^ and CD8^+^ memory T cells [26].

The enhancing effects of IL-15 on memory T cell survival and expansion have been viewed as reasons to investigate the potential vaccine adjuvant effects of this cytokine. Our results showing that significantly increased numbers of TS-specific CD8^+^ T cells persist 6 months after vaccination with pTS plus pIL-15 compared with vaccination with pTS alone (Figure 5) are consistent with the known T cell survival and homeostatic proliferative effects promoted by IL-15. These effects could be due to survival effects triggered in the presence of increased IL-15 during the initial TS-specific T cell activation or maintenance effects provided by prolonged production of IL-15. We were unable to detect increased IL-15 in the serum of mice 6 months post-vaccination with pTS plus pIL-15 (data not shown), but these results do not rule out the possibility that persistence and/or prolonged recycling of surface bound IL-15 occurs on professional antigen presenting cells (e.g. monocytes and/or dendritic cells) expressing the IL-15Rα chain as reviewed above. This would be hard to disprove since IL-15 *trans* signaling has been shown to provide a highly potent mechanism of signaling through both IL-15Rα/IL-15Rβ/γc trimeric and IL-15Rβ/γc dimeric receptor complexes, active with subpicomolar concentrations of IL-15 [25]. Longer-term and more detailed studies are required to evaluate the maximal duration of the enhancing effects of our pIL-15 adjuvant, and to determine the associated period of time of enhanced IL-15 signaling.

In addition to enhancing TS-specific CD8^+^ T cell responses, we report that the pIL-15 adjuvant enhanced TS-specific CD4^+^ T cell responses induced by pTS vaccination (Table 1). Enhanced TS-specific CD4^+^ memory T cell could provide functions in addition to TS-specific CD8^+^ memory T cell responses protective against *T. cruzi* challenges. Enhanced CD4^+^ T cells could provide improved helper function for induction of protective CD8^+^ T cells and/or TS-specific antibody responses capable of targeting the extracellular blood form trypomastigote life stage for destruction. In support of an important role for CD4^+^ T cell responses in the IL-15 enhanced protective effects, we were unable to induce protection in CD4 knockout mice vaccinated with the combination of pTS plus pIL-15 (data not shown). However, enhancement of CD4^+^ T cells alone cannot fully explain the IL-15 enhanced *T. cruzi* protective immunity. We have shown previously that *T. cruzi*-specific CD4^+^ T cells alone cannot transfer protective *T. cruzi* immunity [27].

As mentioned above, enhanced expansion and survival of CD4^+^ memory T cells could provide greater helper effects for TS-specific B cell responses. Alternatively, it is possible that IL-15 could directly enhance antibody responses by signaling through the IL-15Rα known to be expressed on activated B cells [28]. Furthermore, IL-15 has been demonstrated to stimulate proliferation and antibody secretion in B cells facilitated by the inhibition of FAS-mediated apoptosis [29]. However, it seems unlikely that the TS-specific B cell/antibody responses were responsible for the pIL-15-enhanced long-term protection against parasite challenge. We observed potent TS-specific antibody responses 6 months after vaccination in mice given pTS alone, but these mice were not protected against lethal *T. cruzi* challenge. Furthermore, we have shown previously that vaccination of B cell knockout mice with pTS alone confers protection against *T. cruzi* challenge [3]. Thus, antibody responses do not seem to be the likely mediator of enhanced protective immunity induced by IL-15 and TS encoding plasmid vaccines.

Regardless of the possible roles of TS-specific CD4^+^ T cell and antibody responses, the pIL-15-enhanced long-term protective immunity clearly involves important effects on CD8^+^ memory T cells. In fact, we believe the effects of IL-15 on CD8^+^ T cell responses are likely to represent the most important effects responsible for enhancing protective *T. cruzi* immunity. Other investigators have shown that *T. cruzi*-specific CD8^+^ T cells alone can transfer protection [30], [31], and we demonstrate here that TS-specific CD8^+^ T cell responses alone can transfer protective immunity (Figure 6). The addition of pIL-15 to pTS vaccination significantly increased CD8^+^ T cell responses (IFN-γ^+^, TNF-α^+^, IL-2^+^) detectable 6 months post-vaccination (Figure 5 and Table 1). In addition, TS-specific memory CD8^+^ T cells primed by pTS and pIL-15 displayed enhanced lymphoproliferative capacity after antigen re-exposure detectable in CFSE dilution assays (Figure 5C.). Although we were unable to detect differences in memory markers (CCR7, CD62L, and CD127, data not shown) expressed on total and H2K^d^/TS^IYNVGQVSI^-specific T cells obtained from pTS and pIL-15 + pTS vaccinated mice, the combination of enhanced proliferative and effector functions suggests that both central memory (memory T cells with optimal proliferative capacity) and effector memory (expressing optimal immediate effector functions) were improved by the addition of the pIL-15 adjuvant. Further investigations will need to evaluate the relative importance of different subsets of CD8^+^ memory T cell responses for IL-15 enhanced protective *T. cruzi* immunity.

We were impressed by the enormous differences in PMA/ionomycin-induced cytokine responses in both CD4^+^ and CD8^+^ T cells primed by the combination of pTS plus pIL-15 compared with priming by either pTS or pIL-15 alone (Table 1). We detected 4–10 fold increased frequencies of T cells capable of producing IFN-γ, TNF-α, and/or IL-2 after pTS plus pIL-15 vaccination compared with either pTS or pIL-15 given alone. In fact, over 50% of CD4^+^ T cells from pTS plus pIL-15 vaccinated mice produced TNF-α after PMA/ionomycin stimulation (compared with <12% of CD4^+^ T cells from mice vaccinated with pTS or pIL-15 alone). We must emphasize that this remarkable enhancement of polyclonal T cell responses required immunization with both pTS and pIL-15 and was not seen in mice vaccinated only with pIL-15. Total percentages and numbers of CD44^+^ memory CD4^+^ and CD8^+^ T cells were nearly identical in pIL-15 alone, pTS alone and pIL-15 plus pTS vaccinated mice (data not shown), indicating that enhanced cytokine responsiveness in pIL-15 plus pTS vaccinated mice was not due simply to an IL-15-induced expansion of pre-existing polyclonal memory T cells. Overall, these results suggest that in the context of antigen-specific stimulation of T cell responses in vivo, increased levels of IL-15 could result in a marked shift of polyclonal T cells into a more activated state with enhanced responsiveness to inflammatory stimuli. Theoretically, this effect could result in a more broadly reactive epitope spreading of immune responses important for optimal vaccine-induced protection against pathogens with high potential for epitope mutational escape. On the other hand, this increased polyclonal T cell responsiveness could lead to increased risks of autoimmunity or immune-mediated inflammatory disease as adverse effects associated with IL-15 adjuvanted vaccinations or immunotherapies. Future studies will need to explore these possibilities in detail.

In summary, our results demonstrate that a plasmid expressing IL-15 can have significant adjuvant effects for induction of enhanced protective *T. cruzi* immunity and suggest important mechanistic explanations for these effects that should be further analyzed in future research.

## Supporting Information

Figure S1 TS-specific CD4^+^ T cell responses 6 months following pTS±pIL-15 vaccination. BALB/c mice were vaccinated three times, 2 weeks apart with pIL-15, pTS, or pIL-15 + pTS. Six months later, spleen cells (N = 4/group) were cultured overnight with negative control (NC A20) or TS-expressing APC (A20-TS), then ICS performed for quantitation of TS-specific IL-2, IFN-γ and TNF-α producing CD4^+^ T cells. Shown are frequencies of cytokine producing T cells after A20-TS stimulation (gated on CD4^+^ cells, NC A20 stimulation values subtracted). Vaccination with pTS alone or with pIL-15 + pTS induced significantly higher frequencies of IL-2, IFN-γ, and TNF-α-producing CD4^+^ T cells compared to vaccination with pIL-15 alone (* P<0.05 in comparison with pIL-15 alone group).
(0.14 MB TIF)

Figure S2 TS-specific CD8^+^ T cell responses 6 months following pTS±pIL-15 vaccination. BALB/c mice were vaccinated three times, 2 weeks apart with pIL-15, pTS, or pIL-15 + pTS. Six months later, spleen cells (N = 4/group) were cultured overnight with negative control (NC A20) or TS-expressing APC (A20-TS), then ICS performed for quantitation of TS-specific IL-2 and TNF-α producing CD8^+^ T cells. Shown are frequencies of cytokine producing T cells after A20-TS stimulation (gated on CD8^+^ T cells, NC A20 stimulation values subtracted). Vaccination with pTS alone or with pIL-15 + pTS induced significantly higher frequencies of IL-2 and TNF-α-producing CD8^+^ T cells compared to vaccination with pIL-15 alone (* P<0.05 in comparison with pIL-15 alone group).
(0.24 MB TIF)
