# Supplementary figures and images for: Co-Administration of a Plasmid DNA Encoding IL-15 Improves Long-Term Protection of a Genetic Vaccine against Trypanosoma cruzi
Source: PLoS Negl Trop Dis. 2011 Mar 8;5(3):e983. doi: 10.1371/journal.pntd.0000983 (PMC3050911; doi:10.1371/journal.pntd.0000983)

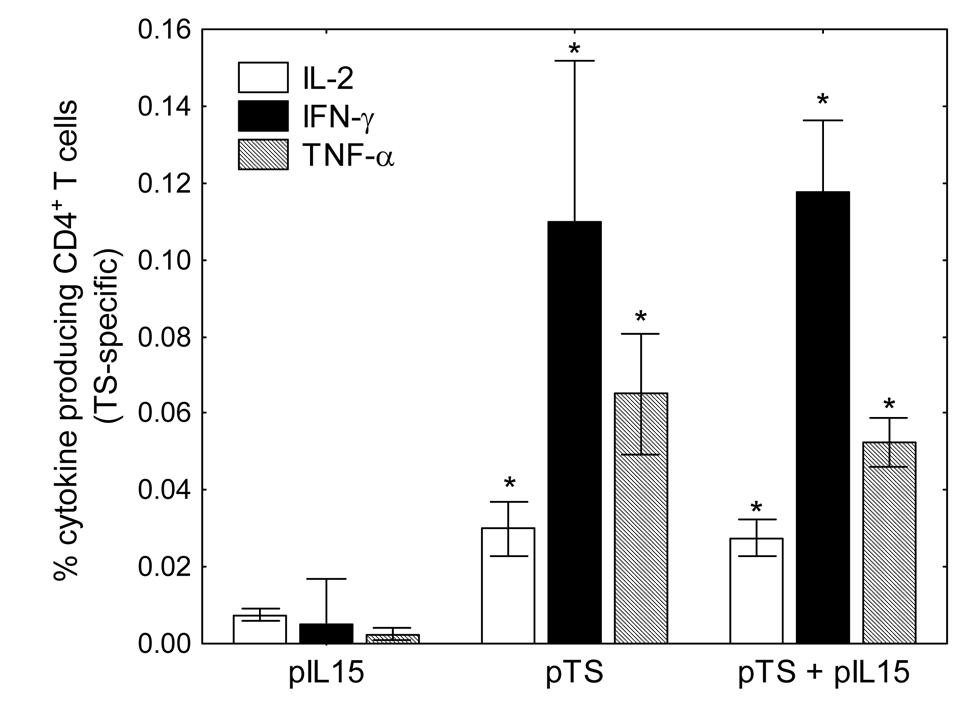

Supplement: Figure S1 — TS-specific CD4+ T cell responses 6 months following pTS±pIL-15 vaccination. BALB/c mice were vaccinated three times, 2 weeks apart with pIL-15, pTS, or pIL-15 + pTS. Six months later, spleen cells (N = 4/group) were cultured overnight with negative control (NC A20) or TS-expressing APC (A20-TS), then ICS performed for quantitation of TS-specific IL-2, IFN-γ and TNF-α producing CD4+ T cells. Shown are frequencies of cytokine producing T cells after A20-TS stimulation (gated on CD4+ cells, NC A20 stimulation values subtracted). Vaccination with pTS alone or with pIL-15 + pTS induced significantly higher frequencies of IL-2, IFN-γ, and TNF-α-producing CD4+ T cells compared to vaccination with pIL-15 alone (* P<0.05 in comparison with pIL-15 alone group). (0.14 MB TIF) [file pntd.0000983.s001.tif]

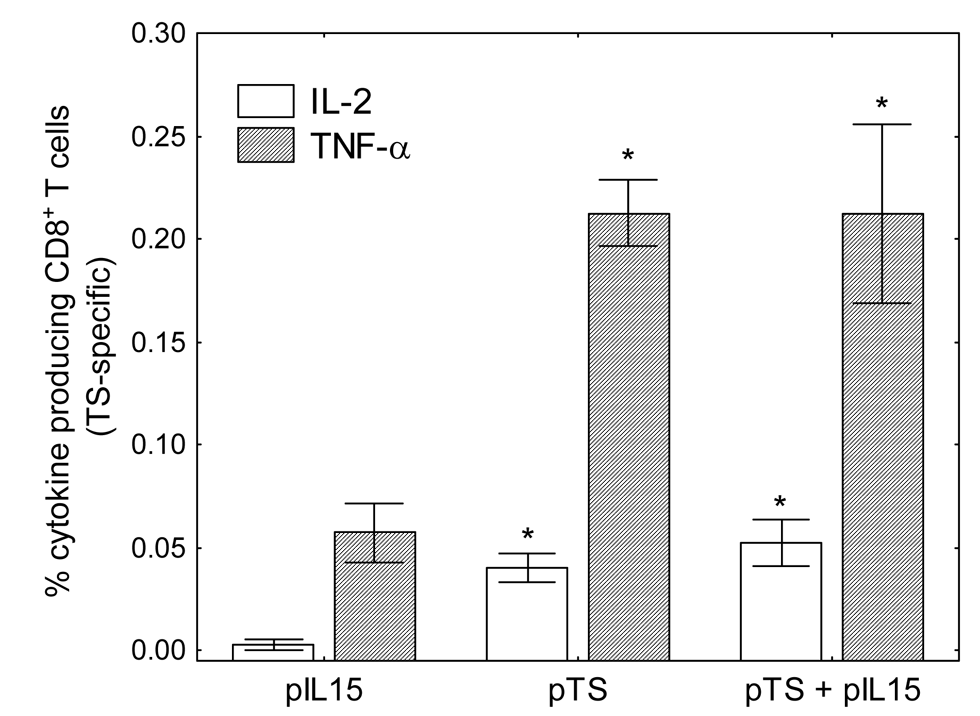

Supplement: Figure S2 — TS-specific CD8+ T cell responses 6 months following pTS±pIL-15 vaccination. BALB/c mice were vaccinated three times, 2 weeks apart with pIL-15, pTS, or pIL-15 + pTS. Six months later, spleen cells (N = 4/group) were cultured overnight with negative control (NC A20) or TS-expressing APC (A20-TS), then ICS performed for quantitation of TS-specific IL-2 and TNF-α producing CD8+ T cells. Shown are frequencies of cytokine producing T cells after A20-TS stimulation (gated on CD8+ T cells, NC A20 stimulation values subtracted). Vaccination with pTS alone or with pIL-15 + pTS induced significantly higher frequencies of IL-2 and TNF-α-producing CD8+ T cells compared to vaccination with pIL-15 alone (* P<0.05 in comparison with pIL-15 alone group). (0.24 MB TIF) [file pntd.0000983.s002.tif]
